# Assisted Reproductive Technology without Embryo Discarding or Freezing in Women ≥40 Years: A 5-Year Retrospective Study at a Single Center in Italy

**DOI:** 10.3390/jcm12020504

**Published:** 2023-01-07

**Authors:** Claudio Manna, Federica Barbagallo, Francesca Sagnella, Ashraf Farrag, Aldo E. Calogero

**Affiliations:** 1 Biofertility IVF and Infertility Center, 00128 Rome, Italy; 2 Department of Biomedicine and Prevention, University of Rome “Tor Vergata”, 00133 Rome, Italy; 3 Department of Clinical and Experimental Medicine, University of Catania, 95123 Catania, Italy

**Keywords:** advanced maternal age, assisted reproductive technique, in-vitro fertilization, oocyte selection

## Abstract

The protocols commonly used in assisted reproductive technology (ART) consist of long-term embryo culture up to the blastocyst stage after the insemination of all mature oocytes, the freezing of all the embryos produced, and their subsequent transfer one by one. These practices, along with preimplantation genetic testing, although developed to improve the live birth rate (LBR) and reduce the risk of multiple pregnancies, are drawing attention to the possible increase in obstetric and perinatal risks, and adverse epigenetic consequences in offspring. Furthermore, ethical–legal concerns are growing regarding the increase in cryopreservation and storage of frozen embryos. In an attempt to reduce the risk associated with prolonged embryo culture and avoid embryo storage, we have chosen to inseminate a limited number of oocytes not exceeding the number of embryos to be transferred, after two days or less of culture. We retrospectively analyzed 245 ICSI cycles performed in 184 infertile couples with a female partner aged ≥40 from January 2016 to July 2021. The results showed a fertilization rate of 95.7%, a miscarriage rate of 48.9%, and a LBR of 10% with twin pregnancies of 16.7%. The cumulative LBR in our group of couples was 13%. No embryos were frozen. In conclusion, these results suggest that oocyte selection and embryo transfer at the cleaving stage constitute a practice that has a LBR comparable to that of the more commonly used protocols in older women who have reduced ovarian reserve.

## 1. Introduction

Louise Brown was born in 1978 with the transfer into the uterus of a single embryo obtained after the laparoscopic retrieval of one oocyte without ovarian stimulation and in vitro fertilization (IVF) [1]. In the following decade, controlled ovarian stimulation (COS) made it possible to collect a higher number of oocytes with the simultaneous transfer into the uterus of more embryos, increasing the success rate of the assisted reproductive technique (ART). Subsequently, embryo freezing became a standard procedure in ART centers to avoid multiple pregnancies. With the development of the vitrification technique, oocyte freezing became possible, and the efficiency of embryo freezing improved.

Another relevant advance in ART is the extended embryo culture up to the blastocyst stage [2]. This procedure has shown a higher implantation potential and a better live birth rate (LBR) in fresh transfer compared to cleaving embryos [3]. However, other studies have shown similar results in cumulative LBR between the two stages of embryo culture [4,5].

Nowadays, a constant trend is to freeze all embryos produced in ART cycles and transfer them one by one—the so-called single embryo transfer (SET)—in a suitably prepared endometrium to reduce multiple pregnancies and maximize the cumulative LBR [6]. Indeed, several studies have shown that SET reduces multiple pregnancies at a rate similar to that of spontaneous pregnancies (3%); in the case of multiple embryo transfer, the rate of multiple pregnancy can be very high (20–50%) [7,8]. Multiple pregnancies are considered the main iatrogenic complication of ART, due to their association with adverse events in both mothers and children [9]. Furthermore, the costs of multiple pregnancies and deliveries are 2–7 times higher than those of singletons [10]. However, not all embryos reach the blastocyst stage (60% for the competence value of IVF laboratories, according to the ESHRE Vienna Consensus of 2017) [11] and in a significant number of cycles the transfer is canceled: 17% in the study of Sainte-Rose et al., especially in older women [12], and 18.8% in the study by De Croo et al. [13]. Furthermore, criticisms have been raised about the effects of the SET policy on the LBR, which does not leave couples the freedom to choose more blastocysts to transfer [14]. Finally, a longer in vitro culture time can be a stressful condition for embryos and can be a source of other possible concerns such as increased obstetric risk [15,16,17,18,19] and epigenetic risk [20,21,22]. Although the negative effects of a long culture on embryos is not fully proven, previous studies reported an increased risk of preterm birth (<37 weeks) [15], small-for-gestational age (SGA) [15] or large-for-gestational age (LGA) [15,19], placenta previa, and placental abruption [15] in pregnancies after blastocyst transfer as compared to pregnancies after cleavage-stage transfer. Furthermore, growing evidence suggests an association between ART and epigenetic modifications that can be transmitted to offspring [23]. Unphysiological conditions, including embryo culture, have the potential to contribute to epigenetic dysregulation [23].

The most challenging endeavor in the ART laboratory is the ability to identify the embryos with the best potential to produce a live birth. The morphological criteria are subjective. Even with “time-lapse” technology, it is not possible to identify with certainty embryos capable of successfully implanting [24]. Preimplantation genetic testing (PGT) is considered useful for discarding aneuploid (PGT-A) embryos to improve the implantation rate and reduce the miscarriage rate [25]. Many studies suggest that PGT-A can implement the use of elective SET (eSET), which selects first-quality embryos based on their morphology for transfer to patients undergoing ART [26], because the combination of the two approaches increases the live birth rate and reduces the multiple pregnancy rate [26]. There is also evidence that PGT-A in women between the ages of 35–40 can improve the clinical and live birth rates, and reduce the negative effects of maternal age on outcomes. However, the cumulative live birth rate does not appear to have improved [27,28]. Indeed, after more than 15 years, this technique still arouses some perplexity [29] because it is not possible to ascertain the ploidy of the whole embryo and, in particular, that of the inner cell mass with a sample of 5–7 cells taken from the trophectoderm. This uncertainty arises from several reasons: the high frequency of embryo mosaicism [30] due to chromosome instability [31], the ability of the embryo to recover from aneuploidy [32,33], and the birth of normal children after the transfer of aneuploid mosaic embryos [34]. Regarding mosaicism, a recent study conducted on 46 surplus cryopreserved preimplantation embryos demonstrated a low rate of cytogenetic concordance (48%) between the inner cell mass and trophectoderm. These results should suggest caution for the clinical application of PGT-A, considering that mosaicism was detected in 59% of embryos (n = 27/46) [35]. Accordingly, a recent meta-analysis showed no significant effect of PGT on the reduction of the miscarriage rate [36]. 

A particular concern in ART practice arises from embryo freezing, which leads to a growing number of embryos stored in cryobanks for an indefinite period of time. Indeed, there was a sharp increase in the United States (U.S.) from 2004 to 2013 of ART cycles in which all embryos have been frozen, and this resulted in a higher and increasing number of embryos stored [37], estimated to be 600,000 (or more) in the U.S. alone [38]. Concern about this topic can arise also in some countries where this practice is not allowed. In fact, in most countries, the mean age of women entering ART programs has seen an increasing trend (34.6% for the ART Italian registry and 20.4% for the U.S. Registry in 2019). Unfortunately, oocyte cryopreservation does not give acceptable results for a woman in the second half of her 30s and, particularly, for patients over 40 [39]. More recently, some authors describe a progression towards the industrialization of ART practices with possible negative consequences for couples such as a decline in ART birth rates [40], although personalized treatments should be a relevant aim for ART [41]. 

In this complex framework that shows possible limits and risks related to ART practices, we report the results of a personalized clinical practice used in a subgroup of women over 40 in our ART center. The protocol consisted of the selection of a limited number of oocytes to be injected, no more than the number of embryos to be transferred, and after a short time of embryo culture (two days or less). The primary aim of this retrospective study was to evaluate the success rate of this protocol in the light of some of the more widespread problems and emerging risks of ART practices.

## 2. Materials and Methods

### 2.1. Patient Selection

This clinical study included 245 ICSI cycles performed from January 2016 to July 2021 in 184 infertile couples with a female partner aged ≥40 years whose clinical charts were evaluated retrospectively. The mean age of the women was 42.4 ± 1.7 (range 40–47 years) and the mean of the previous failed ICSI attempts was 1.5 ± 1.9. The assessment of ovarian reserve was performed through antral follicular count (AFC) which was evaluated by the same experienced gynecologist (CM). 

### 2.2. Controlled Ovarian Hyperstimulation

Controlled ovarian hyperstimulation protocols were performed using recombinant human follicle-stimulating hormone (rhFSH) (Gonal-F, Merck Serono, Geneva, Switzerland) according to ovarian reserve, and gonadotropin-releasing hormone (GnRH) antagonist (0.25 mg) from the day when a follicle reached 15 mm in diameter.

We also administered recombinant human luteinizing hormone (LH) at 75 IU (Luveris, Merck Serono, Geneva, Switzerland) every 12 h along with rhFSH increased by 75 IU during GnRH administration, according to data showing better outcomes in older female patients [42] and dramatic decrease in serum LH as a consequence of GnRH antagonist administration [43]. Follicular development monitoring was performed by real-time ultrasound scans from day 2 of the treatment cycle to the day of hCG administration based on the patient’s response to stimulation. The response was monitored by ultrasound, and measurements of serum levels of 17ß-estradiol, progesterone, and FSH, including on weekends or holidays. When at least one ovarian follicle reached a diameter of 18–20 mm, ICSI was performed 36–38 h after administration of human chorionic gonadotropin (hCG, Gonasi, 10,000 IU) (IBSA, Lodi, Italy). 

### 2.3. Oocyte Retrieval

Oocyte retrieval was scheduled on a 7-day basis and performed with local analgesia or under sedation 36–38 h after hCG administration based on response to ovarian stimulation. 

### 2.4. Sperm Preparation

The first semen collection was obtained approximately 5–6 h before the microinjection of oocytes, which was scheduled approximately 40 h after the hCG administration to the female partner. All male partners had 2–7 days of abstinence, as suggested by the WHO 2010 criteria [44]. All semen samples were collected by ejaculation within the Fertility Center to minimize conditions that could alter sperm parameters/function. All semen analyses were performed by the same expert embryologist according to WHO 2010 criteria. The assessment of sperm motility was performed on a 10 μL drop on a slide with a 22 × 22 mm coverslip and a stage heated to 37 °C, with a reticule lens. The slides were examined with phase-contrast optics at a magnification of 400×. We evaluated 400 spermatozoa per replicate for an accurate assessment of motility. We asked male partners with severe oligoasthenozoospermia (OA) to provide a second consecutive ejaculation 1 h after the first, after explaining the possibility of having better sperm parameters in the second ejaculate [45,46].

### 2.5. Intracytoplasmic Sperm Injection Procedure

The ICSI procedure was performed with spermatozoa obtained by “swim-up” using the first or second ejaculate according to the sperm parameters of the male partner as described in Section 2.4. The “swim-up” technique was performed directly from the liquefied semen. For this purpose, several aliquots of semen were taken from each sample and placed in test tubes underneath an overlay of washing medium (Origio Italia Srl, Rome, Italy). Round-bottom tubes or four-well dishes were used to optimize the interface surface area between the semen layer and the culture medium. The samples were allowed to incubate at 37 °C in an incubator for 30–45 min. Spermatozoa with the best motility and ability to migrate were then collected. 

Collected cumulus-enclosed oocytes were maintained in 500 µL of Continuous Single Culture™ Medium-Complete (CSCM-C) (Irvine Scientific, FujiFilm, Tilburg, the Netherlands) in 4-well multi-dishes (Nunclon Surface, Roskilde, Denmark) under oil (oil for embryo culture, Fuji Film, Europe), and maintained in the incubator for 2 h after their retrieval. Afterward, they were decumulated in hyaluronidase drops (Hyaluronidase Solution, Fuji Film, Europe). The ICSI procedure was performed according to the standard technique. 

### 2.6. Selection of Oocytes and Transfer Policy

The choice of the oocyte to be inseminated was made after decumulation. The best oocytes to inseminate were those with the following characteristics: small perivitelline space and no granulation [47], intact first polar body (PB) [48,49], and a smooth surface [50]. We discarded oocytes with vacuolar cytoplasm or central granulation, ovoid-shaped formation [51], cytoplasmic inclusion [48], smooth endoplasmic reticulum (SER) aggregates [52], and refractive bodies [53]. Oocyte selection was also based on oolemma elasticity, a parameter that positively influences the outcome of ICSI. In particular, we have distinguished three different degrees based on the elasticity of the oolemma: grade A refers to oocytes that have penetrated the oolemma without the need for cytoplasmic aspiration (no elasticity); grade B refers to oocytes showing oolemma penetration requiring mild or moderate cytoplasmic aspiration (average elasticity); grade C refers to oocytes showing oolemma penetration requiring strong cytoplasmic aspiration (excessive elasticity). If no oocyte reached the highest grade, which is grade B, the closest grade was chosen based on the oolemma characteristics (grade C and, lastly, grade A). 

Embryo culture was performed in a standard incubator at 37 °C under 6% CO_2_ and 5% O_2_ in CSCM-C (Irvine Scientific, FujiFilm, Tilburg, The Netherlands). Embryo transfer was usually performed after 2 days of culture. In some cases, the transfer was performed at the pronuclear stage. After 36–44 h of culture, all embryos were carefully examined with both a dissecting and an inverted microscope. The classification of the embryos was carried out according to the system proposed by Puissant [54]. The number and size of blastomeres as well as the presence or absence of anucleated fragments were carefully recorded so that embryos could be scored as follows: 4 = embryos with clear and regular blastomeres and no fragmentation or a maximum of five % of the embryo surface occupied with small anucleated fragments; 3 = embryos with few or no fragments but with unequal blastomeres (>1/3 difference in size); 2 = embryos with more fragments but less than 1/3 of the embryo surface; 1 = fragments on >1/3 of the embryo surface. Two points were added if the embryo had reached the 4-cell stage by 48 h after fertilization. 

With regard to the maximum number of embryos to be transferred, we followed the guidelines of the Practice Committee of the American Society for Human Reproduction and Society for Assisted Reproductive Technology [9]. Therefore, in patients aged 40 years, three or four embryos could be transferred in the case of a particularly unfavorable prognosis; in patients aged 41–44 years, four embryos could be transferred, or even five when an unfavorable prognosis was present. A prognosis was considered unfavorable in the case of multiple previous ART cycle failures or no live births after an ART cycle. The informed consent was signed by the couples after an extensive discussion with the physicians on the maximum number of oocytes to be inseminated and, consequently, of embryos to be transferred. Eleven oocytes belonging to three couples were cryopreserved at their express request. In all cases, the selection of oocytes for the transfer of the resulting embryos was carried out according to the described criteria.

### 2.7. Ethical Approval 

The study was conducted in the ART “Biofertility IVF Center” (Rome, Italy) on infertile couples undergoing ICSI treatment. It was reviewed and approved by the Institutional Review Board at the “Biofertility IVF Center”, which indicated that ethical approval was not required for this study. Data collection followed the principles outlined in the Declaration of Helsinki. All patients provided their informed consent, agreeing to supply their anonymous information for this and future studies. 

### 2.8. Statistical Analysis 

Quantitative data were reported as mean ± SD throughout the study. The following rates were calculated: fertilization rate (FR = number of fertilized oocytes/number of oocytes inseminated), implantation rate (IR = number of gestational sacs/number of embryos transferred), clinical pregnancy rate (CPR = number of pregnancies with at least one fetal heartbeat/number of pick-up cycles with at least one oocyte retrieved), live birth delivery rate (LBR = number of deliveries with at least 1 live birth/number of pick-up with at least 1 oocyte retrieved), miscarriage rate (MR = number of spontaneous abortions/total number of pregnancies), and cumulative live birth rate (CLBR = number of deliveries with at least 1 live birth/total number of women with aspirated oocyte(s)) were calculated. Data were analyzed with SPSS 23.0 for Windows (SPSS Inc., Chicago, IL, USA).

## 3. Results

A total of 245 ICSI cycles performed in 184 couples with female partners aged ≥40 years were considered. Among the 184 couples enrolled, 39 couples underwent two ICSI cycles, 7 couples three attempts, and 2 couples four cycles. 

Table 1 shows the clinical and demographic characteristics of the couples enrolled in this study and their previous failed ICSI attempts, which include both attempts performed in our and other ART centers. In six cycles, no oocytes were retrieved. Considering the repeated attempts for each couple, only two women had no transfer. Therefore, 182 women aged ≥40 years underwent 239 cycles with oocyte retrieval and embryo transfer. A total of 705 embryos were transferred with a mean number of 2.9 ± 1.4 embryos per transfer. In 35 cycles, the embryos were transferred at the pronuclear stage.

The outcomes of ICSI cycles are shown in Table 2. 

**Figure 1:**
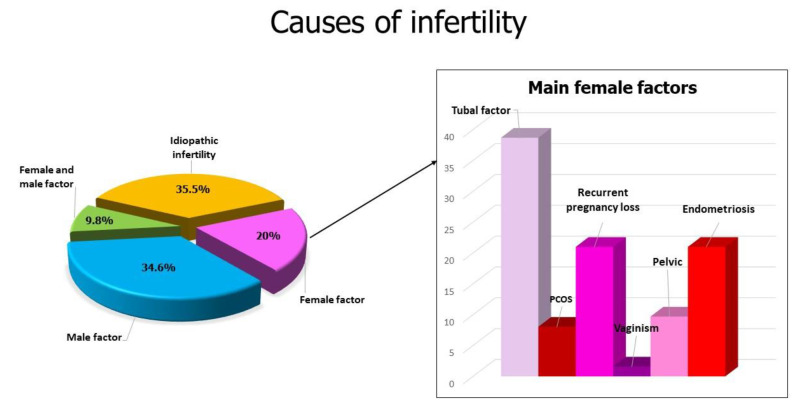
(**Left panel**): Main causes of infertility found in the 184 couples enrolled in this study. (**Right panel**): Detail of the main causes of female infertility.

Twenty-four women have had at least one pregnancy. All pregnancies occurred in women between the ages of 40 and 44 years. As reported in the Materials and Methods section, according to the guidelines of the Practice Committee of the American Society for Human Reproduction and Society for Assisted Reproductive Technology [9], we transferred a maximum of five embryos when an unfavorable prognosis was present. In total, we transferred five embryos in 48 cycles of our cohort. Interestingly, all pregnancies occurred when at least three embryos were transferred, except in five cases where two embryos were transferred. In detail, if we consider the 24 cycles with pregnancy leading to delivery and live birth: in half of them (12 cycles), five embryos were transferred; in five cases, four embryos were transferred; in two cases, three and in five cases, two. Of the 24 pregnancies, four were twins; in three of those cases, five embryos were transferred, while in one case five embryos were transferred. No triplets occurred. In Table 3, we report the number and grade of embryos transferred and the related success rates. Four pregnancies with deliveries occurred among 35 cycles with embryo transfer at the pronuclear stage, with a LBR of 11.4% (4/35), and included the two cycles with women at 44 years ending with normal delivery. Table 4 presents the LBR based on the age of the women. The twenty-four women who achieved pregnancy had a previous mean failure of 1.9 ± 1.8. The causes of infertility of the couples enrolled are reported in Figure 1.

## 4. Discussion

The results of the present study indicate that the selection of oocytes before ICSI to obtain a predetermined number of fresh cleaved embryos to be transferred is effective in terms of FR and LBR in a group of women ≥40 years. Our delivery rate (number of monitored deliveries/number of pick-ups or DR) was not lower than that of the Italian registry for the same age group (4.7% = 728/15,419) published in 2019 and not far from all age groups (11.2% = 5151/46,090) [55]. Furthermore, we reported a cumulative delivery rate (CLBR) for fresh cycle of 13%, whereas the Italian registry reported a CLBR of 5.9% for women aged between 40–42 years and 1.6% for women aged ≥43 years. With regard to CLBR per pick up, the Italian registry listed 10.3% for women aged between 40–42 years and 3.2% for women aged ≥ 43 years, whereas the U.S. registry reported a cumulative transfer live birth delivery (LBD) rate per pick-up of 13% in 2019 [56]. It should be noted that the CLBR with frozen embryos rules out cycles unable to give enough embryos for freezing procedures. 

With regard to the success rate of blastocyst transfer in aged women, Tannus and colleagues reported a higher LBR than ours (21.6%), although the mean AFC was 14, mean of previous failed cycles was 0.5, and oocytes collected was 11 [57]. Our patient group exhibited a less favorable AFC (8.4) and a higher number of previous failed ART cycles (1.5 ± 1.9) (Table 1). In another study conducted by De Croo and colleagues, in women with a mean age of 35 years, the comparison of LBRs with transfer at the blastocyst stage (1 or 2 embryos) versus cleaved embryos was 21.1% and 19.1%, respectively [13].

A specific and usual reason for long-term embryo culture up to the blastocyst stage is to perform PGT-A. Apart from the reduced rate of blastocyst formation in patients over 40 years of age, several concerns have been raised for the PGT-A technique, such as the high genetic mosaicism rate, which interferes with the precise evaluation of embryo chromosomal arrangement and the mismatch in the aneuploidy rate between the trophectoderm and the inner cell mass [29]. Furthermore, increased obstetric and perinatal risks are reported with PGT-A compared with non-PGT-A cycles, particularly the development of hypertension in pregnancy [58]. However, PGT-A has become the most widely utilized add-on procedure in ART practice [29] and is a reference for validating or at least comparing the results of many clinical trials in the U.S.

One of the possible advantages of reconsidering embryo transfer in the cleavage stage is the epigenetic risk after embryo exposure to a long culture environment in terms of fetal health. Many studies have shown that extended embryo culture significantly affects obstetric and perinatal outcomes [19,59,60,61]. Large-for-gestational age/macrosomia, hypertensive disorders, and perinatal mortality appear to increase with frozen embryo transfer [62]. Vroman and colleagues have demonstrated that embryo culture from the one-cell to blastocyst stage results in placental overgrowth, reduced fetal weight, and lower placental DNA methylation in rats [63]. Surprisingly, a recent study demonstrated that human genomic activation initiates at the one-cell stage [64]. 

There is evidence that the longer the in vitro cultures last (i.e., blastocyst transfer in comparison to the cleavage stage), the more epigenetic changes occur [65,66]. We know that only a percentage of fertilized oocytes arrive at the blastocyst stage in vitro and recent observations suggest that metabolic and epigenetic dysfunctions underlie the arrest of human ART embryos before their compaction [67].

From a biological point of view, we cannot rule out the existence of better “culture” conditions for embryos in uterus rather than in vitro (temperature, pH, osmolarity, and numerous unknown factors). Interestingly the LBR subsequent to transfer at the pronuclear stage (at 44 years, two of them delivered at term without obstetric or perinatal complications) does not seem negligible (11.4%), supporting the idea that an artificial incubator environment might be more stressful than that within the uterus for embryos in older women. Most of the studies on obstetric and perinatal risk in ART are linked to placental abnormalities that are increased when ART is the chosen treatment of infertility, particularly in more stressful conditions for embryos, such as long-term cultures and PGT-A [68].

Concerns about the possible consequences of ICSI for the health of the offspring have been reported since its first introduction into clinical practice [69]. Although ICSI use was associated with a significantly higher risk of congenital malformations [70], other studies did not report a significant difference in terms of congenital malformation between children conceived with IVF/ICSI compared to natural conception [71,72]. A recent systematic review and meta-analysis showed no differences in the epigenetic effects of offspring between couples treated with ICSI or traditional IVF [73]. In summary, after many decades of ICSI practice and according to most reports, children born after ICSI have perinatal outcomes comparable to those conceived after standard IVF.

With regard to the possible success rate of oocyte selection in ART practice, in 2004 and for many years, the Italian ART legislation limited the maximum number of oocytes to be fertilized during an ART cycle to three, and all resulting embryos had to be transferred at once due to the banning of embryo cryopreservation [74]. Ragni and colleagues, in a study including 1861 cycles performed in seven Italian fertility centers, showed that the pregnancy rate per oocyte retrieval and the rate of multiple pregnancies before and after the new law were 27 and 24.2% (*p* = 0.18), and 25.8 and 20.9% (*p* = 0.11), respectively [68]. It is worth noting that in countries such as Germany and Switzerland, it is impossible to cryopreserve embryos and selection is based on oocytes at the pronuclear stage.

Regarding the problem of twin pregnancy rate, our results (16.7%) appear acceptable, considering that the Italian registry showed a rate of 10.6% in 2019 and it was 16.9% in Europe [75]. Recent reports criticize the SET policy in favor of double embryo transfer at the blastocyst stage [6]. However, our transfers were performed at the cleavage stage, in which a higher number of embryos transferred is to be considered comparable with a lower number at the blastocyst stage. Considering the risk of twin or multiple-order pregnancies and their resultant cost, the results of our study should be taken with caution for general clinical practice. Indeed, as reported in Table 3, in a considerable number of cycles we decided to transfer three or more embryos because there was a poor prognosis. At present, insemination with the transfer of more than two embryos should not be a routinely offered practice, even though the latest U.S. guidelines allow the transfer of more than two embryos for older women, low-quality embryos, and repeated implantation failures. On the other hand, blastocyst transfer is associated with a higher risk of monozygotic twinning (MZT) [76] which has a more severe prognosis than dizygotic twinning for the risk of twin-to-twin transfusion due to their shared placenta. After 8435 frozen-thawed single blastocyst transfers with hormone replacement treatment, MZT was observed in 2.32% of cases [77], while the natural prevalence was 0.4% [78]. However, the transfer of a very limited number of embryos at their cleavage stage after insemination of selected oocytes may represent a practical option in cases of high risk for twin or multiple pregnancies. 

Clinical trials with a mix of oocyte selection and embryo selection at the cleavage stage may be considered, even in couples with female partners under the age of 40. It could represent a kind of double selection in order to simultaneously reduce the obstetric/epigenetic risk and the risk of multiple pregnancies.

Regarding the efficiency of oocyte selection, we notice that our FR was higher (95.7%) compared with the usually reported data such as that of ESHRE/Alpha consensus (≥65% for competence value) [11], probably due to a selection of oocytes based on multiple morphologic elements studies [48,49,79]. 

We recognize that oocyte selection in ART is mostly still imperfect, mainly because it is subjective. However, with the introduction of artificial intelligence in ART, new tools may be available to promote more objective observations [80,81], as we have previously proved [82]. Nevertheless, we should not forget that even the current embryo selection is also a subjective laboratory procedure. The possible transition from embryo to oocyte selection, using more reliable methods, could provide us with valuable information on the relationship between oocyte quality and stimulation protocols and, consequently, embryo development.

In summary, our study showed the success rate and twin delivery rate in women over the age of 40 using a protocol with oocyte (rather than embryo) selection and transfer of embryos in the cleavage stage. Non-negligible LBR and moderate multiple pregnancy rates were recorded. No embryos were frozen.

The application in clinical practice of the results described in the present study can be relevant for geographical areas where embryo freezing is not possible for ethical reasons or law restrictions, or for couples with low prognosis with a female partner aged ≥40 who do not accept oocyte donation.

Furthermore, the financial implications and cost/benefits of this protocol, i.e., strong personalization and drug use, are to be considered. In this regard, milder stimulation for this group of patients based on their residual ovarian reserve could offer similar chances of success. The small size of this subgroup of patients undergoing ART and the lack of a control group are the main limitations of our study. However, we enrolled a particular group of couples with female partners aged ≥40 years, which made it difficult to establish a control group. Certainly, further prospective randomized controlled trials are needed to assess the relevance of our retrospective findings.

## 5. Conclusions

In conclusion, the possibility of oocyte selection and embryo transfer at the cleavage stage appears to be a reasonable strategy in older women who have reduced ovarian reserve and a high number of previous ART failures. As clinicians, we should consider current trends in reproductive medicine from a broad perspective, taking into account all possible consequences involving obstetricians, neonatologists, pediatricians and all other professionals interested in the long-term health consequences of ART laboratory practice. Although further studies are needed to confirm these findings, our preliminary results suggest that a return to more natural steps in reproductive medicine may be safer if the obstetric risks and epigenetic consequences on offspring linked to long-term culture protocols are confirmed in the future.

## Figures and Tables

**Table 1:** Demographic and clinical characteristics of the female and male partners of the couples enrolled in this study.

Parameters	Results
Women	
Age (years, mean ± SD)	42.4 ± 1.7
Antral follicle count (mean ± SD)	8.4 ± 4.9
Total dosage of gonadotropin administered (IU) (mean ± SD)	3376.6 ± 1335.9
Men	
Age (years, mean ± SD)	43.9 ± 5.9
Sperm concentration (mil/mL, mean ± SD)	46.7 ± 40.6
Total sperm motility (%, mean ± SD)	47.4 ± 21.3
Progressive sperm motility (%, mean ± SD)	26.3 ± 16.8
Spermatozoa with normal morphology (%, mean ± SD)	15.2 ± 10.3
Couples	
Number of previous ART failures (mean ± SD)	1.5 ± 2

Legend: IU: international unit.

**Table 2:** Intracytoplasmic sperm injection outcomes.

Fresh cycles	245
**Number of oocytes retrieved** (mean ± SD)	5.2 ± 4.1
**Number of oocytes inseminated** (mean ± SD)	3.1 ± 1.5
**Number of oocytes fertilized** (mean ± SD)	3.1 ± 1.5
**Number of oocytes cryopreserved** (mean ± SD)	0.06 ± 0.5
**Peak of stimulated 17ß-estradiol** (pg/mL)	1271.0 ± 744.5
**Fertilization rate** Number of fertilized oocytes/number of oocytes inseminated (%)	715/747 (95.7%)
**Implantation rate** Number of gestational sacs/number of embryos transferred (%)	47/705 (6.7%)
**Clinical pregnancy rate** Number of pregnancies with least 1 fetal heartbeat/number of pick-up cycles with at least 1 oocyte (%)	31/239 (13%)
**Live birth delivery rate** Number of deliveries with at least 1 live birth/number of pick-ups with at least 1 oocyte (%) Singletons Twins	24/239 (10%) 20 4 (16.7%)
**Birth weight** (g, mean ± SD)	3112.3 ± 698.9
**Miscarriage rate** Number of spontaneous abortion/total number of gestational sacs (%)	23/47 (48.9%)
**Cumulative live birth rate** Number of deliveries with at least 1 live birth/total number of women starting treatment (%)	24/184 (13%)

**Table 3:** Number and grade of embryos transferred and related success rates.

Number of Embryos Transferred	Total Number of Embryo- Transfers	Embryos Grade I	Embryos Grade II	Embryos Grade III–IV	PN	Live Births	Twin Birth	Multiple Births (≥3)
1	n = 42 (17.6%)	n = 23	n = 10	n = 4	n = 5	n = 0	n = 0	n = 0
2	n = 59 (24.7%)	n = 69	n = 32	n = 1	n = 16	n = 5	n = 0	n = 0
3	n = 55 (23.0%)	n = 97	n = 39	n = 5	n = 24	n = 2	n = 1	n = 0
4	n = 35 (14.6%)	n = 91	n = 27	n = 5	n = 17	n = 5	n = 0	n = 0
5	n = 48 (20.1%)	n = 156	n = 54	n = 14	n = 16	n = 12	n = 3	n = 0

Legend: grade I = blastomeres of equal size, without fragmentation or with <10% fragmentation; grade II = slight asymmetry between blastomeres and fragmentation of 10–25%; grade III–IV = asymmetric blastomeres and fragmentation of ≥35%. Abbreviations: PN = pronuclear stage.

**Table 4:** Live birth rates based on the woman’s age and the number of cases (n).

Age of Women (Years)	Number of Live Births (%)
40 (n = 33)	7/24 (29.2%)
41 (n = 53)	6/24 (25%)
42 (n = 51)	5/24 (20.8%)
43 (n = 42)	4/24 (16.7%)
44 (n = 39)	2/24 (8.3%)
≥45 (n = 27)	0/24 (0%)
